# The inhibitory effect of sense of power on experiential sports consumption: the chain-mediated role of psychological distance and social orientation

**DOI:** 10.3389/fpsyg.2025.1590197

**Published:** 2025-05-14

**Authors:** Kai Guo, Qian Huang

**Affiliations:** ^1^School of Economics and Management, Wuhan Sports University, Wuhan, Hubei, China; ^2^College of Intelligent Sports Engineering, Wuhan Sports University, Wuhan, Hubei, China

**Keywords:** experiential sports consumption, sense of power, psychological distance, social orientation, sports consumer behavior

## Abstract

**Introduction:**

To explore the internal driving mechanisms influencing consumers' preferences for experiential sports consumption, this study constructs a structural equation model with experiential sports consumption as the dependent variable and sense of power as the independent variable, integrating the Agentic–Communal Model of power perception. Psychological distance and social orientation are introduced as mediating variables.

**Methods:**

This study utilized survey data from 1,062 Chinese sports consumers to empirically test the proposed structural equation model.

**Results:**

The analysis reveals the following findings: 1) Sense of power has a significant negative predictive effect on experiential sports consumption, with consumers perceiving lower sense of power more likely to choose experiential sports consumption; 2) Psychological distance mediates the relationship between sense of power and experiential sports consumption. Lower sense of power makes consumers feel a closer psychological distance, which in turn makes them more inclined to choose experiential sports consumption; 3) Social orientation also plays a mediating role in this process, with consumers perceiving lower sense of power more likely to seek social interaction to enhance their consumption experience through higher social orientation; 4) Psychological distance and social orientation jointly form a chain mediation effect between sense of power and experiential sports consumption. Lower sense of power shortens psychological distance, which in turn enhances social orientation, thus driving the choice of experiential sports consumption.

**Discussion:**

The research findings not only expand the theoretical framework of the impact of sense of power on sports consumer behavior but also provide theoretical support for sports enterprises when developing differentiated marketing strategies, particularly in how to design personalized strategies based on consumers' perceived differences in sense of power.

## 1 Introduction

In recent years, with the continued economic development and the improvement of living standards, the significance of the sports industry within the global economic system has become increasingly prominent (Wu, 2024). In China, the sports industry has shifted from a traditional focus on scale expansion to one centered on high-quality development, emphasizing quality and efficiency. This transformation has facilitated the optimization of industrial structure and profoundly influenced consumer behavior in sports consumption (Li et al., 2025). Notably, the structure of sports consumption has undergone a significant shift, with experiential sports consumption gradually replacing tangible consumption as the dominant market trend (Li and Qu, 2025). This change reflects the growing demand for diversified and personalized sports experiences, while also providing direction for the future development of the sports industry. Understanding the driving forces behind this shift in consumption structure not only helps reveal the psychological underpinnings of consumer behavior in contemporary society but also provides important theoretical and practical insights for the sustainable growth of the sports industry.

In contrast to traditional tangible sports consumption, experiential sports consumption has become a crucial component of the sports market due to its unique emphasis on participation, experience, and social interaction (Matte et al., 2024). Experiential sports consumption emphasizes the subjective experience and emotional satisfaction of consumers during the consumption process (Chanavat and Bodet, 2014), such as building social connections through participation in sports activities or shared viewing experiences (Zhou and Kaplanidou, 2018). The rise of this consumption model has not only injected new vitality into the sports industry but also posed new challenges for consumer behavior research. Previous studies have primarily explored experiential sports consumption from a macro perspective, focusing on policy support or industrial characteristics, with limited attention given to the antecedent variables of consumer preferences at the micro level, particularly the psychological factors that are prevalent in everyday life. Identifying the psychological mechanisms that drive experiential sports consumption and clarifying the differences between these and preferences for tangible consumption have become urgent academic challenges. This study addresses this issue by focusing on a key psychological variable—sense of power—and aims to reveal its impact on sports consumption behavior and underlying mechanisms.

Sense of power, as a core psychological variable that reflects an individual's perception of their ability to influence and control others, has been shown to have a profound effect on purchase decisions and preferences in consumer behavior research (Guinote, 2017; Rucker et al., 2012). Specifically, consumers in a low-power state are more likely to purchase symbolic goods to compensate for their perceived lack of status and power (Rucker and Galinsky, 2008), while consumers in a high-power state are more concerned with the functional value of products, leading to a preference for utilitarian consumption (Rucker and Galinsky, 2009). From a cognitive perspective, individuals with a high sense of power tend to focus on themselves (self-focus; Miyamoto and Ji, 2011), are more likely to purchase products for themselves (Rucker et al., 2011), and exhibit a higher tendency to save (Garbinsky et al., 2014). Conversely, individuals with a low sense of power are more prone to focus on others (other-focus; Miyamoto and Ji, 2011), preferring products with altruistic appeals (Rucker et al., 2012), and are more likely to engage in impulse buying (Song et al., 2023). Given these differences in cognitive tendencies, can these power-induced differences help predict sports consumers' preferences for either tangible or experiential sports consumption? This study seeks to address this question by exploring the impact of sense of power on sports consumption behavior (tangible vs. experiential) and its underlying mechanisms.

As an important social activity, engaging in sports consumption experiences has long served as a means for sports consumers to establish behavioral and emotional connections with others (Raghunathan and Corfman, 2006). Due to the social nature of experiential consumption, participating in experiential sports consumption fulfills relational and belonging needs, and its underlying mechanisms may be influenced by factors such as social interaction, social connections, and social distance (Guo et al., 2024). Thus, sense of power can induce changes in multiple intrinsic psychological needs, while experiential sports consumption, with its experiential characteristics, can fulfill various psychological needs. Which psychological needs serve as crucial bridges linking sense of power with preferences for tangible or experiential sports consumption? This is another question this study aims to address through the development of a conceptual model.

This research aims to empirically analyze the negative impact of sense of power on experiential sports consumption and explore the mediating roles of psychological distance and social orientation in this process. The findings will not only deepen the understanding of sports consumer behavior and enrich the theoretical framework of consumer psychology, but also provide practical guidance for sports enterprises in developing differentiated marketing strategies.

## 2 Literature review and research hypotheses

### 2.1 Definition of experiential sports consumption and current research status

In recent years, academic discussions have generally classified sports consumption into two categories: tangible sports consumption and experiential sports consumption (Li and Qu, 2025). Experiential sports consumption can be further subdivided into participatory forms (e.g., fitness training) and spectator forms (e.g., attending sporting events; Jeon et al., 2021; Kim and James, 2019). This classification stems from the classic distinction between experiential and tangible consumption made by Van Boven and Gilovich (2003). Specifically, tangible sports consumption refers to behaviors aimed at acquiring tangible, possessable sports products (such as sports apparel or equipment), emphasizing “ownership and preservation,” while experiential sports consumption focuses on acquiring intangible experiences (such as sports tourism or leisure spectating), emphasizing “experiencing and feeling” (Guo et al., 2024; Matte et al., 2024).

Compared to tangible sports consumption, experiential sports consumption offers a more pronounced enhancement of the consumer's hedonic experience and has a stronger effect on pleasure, satisfaction, and overall happiness (Gilovich et al., 2015; Kim and James, 2019). The underlying reason for this lies in the inherently social nature of experiential sports consumption, such as sports tourism, spectating, event participation, and outdoor mountain activities, which fosters interpersonal interaction (Guo et al., 2024; Zhou and Kaplanidou, 2018). This type of consumption not only promotes social relationships but also satisfies individuals' need for connection, thus enhancing their overall sense of wellbeing (Dunn et al., 2011; Guo et al., 2024). It is worth noting that the rapid development of digital technology has given rise to new forms of experiential sports consumption. For example, esports live streaming builds a sense of virtual community belonging through real-time interaction via bullet comments (Hamari and Sjoblom, 2017), while virtual reality (VR) technology breaks the spatial limitations of traditional offline experiences by simulating immersive sports scenarios (Neumann et al., 2018). These digital forms not only preserve the social core of traditional experiences but also create new dimensions of interaction through technological empowerment, further amplifying consumers' emotional involvement (Hadi et al., 2024). Additionally, social comparison theory suggests that experiential sports consumption, due to its subjectivity and uniqueness, is less likely to provoke direct comparisons with others, thereby reducing anticipated regret and negative emotions (Rosenzweig and Gilovich, 2012). Furthermore, experiential sports consumption can transform into lasting memories, disseminated through narratives, and endow sports consumers with a deeper sense of meaning (Guo et al., 2023; Nicolao et al., 2009). For instance, the excitement of victory or the disappointment of defeat during event spectating often become unforgettable experiences, far surpassing the value of tangible products. Thus, experiential sports consumption has gradually emerged as a highly valued consumption form among sports consumers, making the exploration of its influencing factors and mechanisms of action particularly important.

In the research related to experiential consumption, various factors have been widely recognized for their positive impact on experiential consumption, including feelings of loneliness (Zhao and Jin, 2021), social needs (Gilovich et al., 2015), peer participation (Zeng et al., 2024), and social class (Lee et al., 2018). Jeon et al. (2021) emphasized that in sports fitness consumption, the service environment significantly enhances consumer satisfaction and loyalty by influencing emotional experiences and spatial flow, with knowledge acquisition motives playing a moderating role (Jeon et al., 2021). In the research on spectator sports consumption, Wang et al. (2024), through grounded theory analysis, identified four dimensions—individual factors, psychological factors, situational factors, and product factors—that jointly influence the public's sports consumption intentions (Wang et al., 2024). Matte et al. (2024) pointed out that leisure participation and voluntary simplification positively influence leisure satisfaction and experiential consumption (Matte et al., 2024). In addition, differences in cultural values have been found to influence preferences for experiential consumption. For example, consumers in high-power-distance cultures are more likely to strengthen social bonds through group activities (e.g., group spectating, family sports tourism), while individuals in low-power-distance cultures are more likely to focus on autonomous expression of personalized experiences (Zhang et al., 2008; Zheng and Shen, 2025).

Although experiential sports consumption has become a hot topic in academic research, there remains a lack of exploration into the underlying psychological mechanisms of consumers. In particular, how psychological traits such as sense of power, psychological distance, and social orientation influence preferences for experiential sports consumption has not yet been systematically examined in the literature. Therefore, this study aims to empirically analyze the mechanisms through which consumer psychological characteristics affect preferences for experiential sports consumption. By doing so, this research seeks to enrich the theoretical framework of experiential sports consumption and contribute to a more nuanced understanding of consumer behavior.

### 2.2 Sense of power and research hypothesis on experiential sports consumption behavior

Power is generally defined as the asymmetric control over valuable resources within social relationships (Magee and Galinsky, 2008), while sense of power refers to an individual's subjective perception of their influence and control (Anderson et al., 2012). Sense of power not only shapes an individual's cognitive processes and interpersonal interactions but also profoundly impacts their behavioral decisions. The Agentic–Communal Model emphasizes that sense of power influences an individual's judgment in the process of weighing self-importance (Rucker et al., 2012). High sense of power fosters an agentic orientation, where individuals perceive themselves as relatively independent and unrestrained by external environments, thereby focusing more on self-expression, self-enhancement, and self-protection (Miyamoto and Ji, 2011). In contrast, low sense of power fosters a communal orientation, where individuals are perceived as warmer, more submissive, and less capable, leading them to focus more on others, rely on others, and value others' feelings (Rucker and Galinsky, 2016).

In recent years, the psychological effects of sense of power in consumer behavior have received significant attention. Research on impulsive consumption suggests that consumers with high sense of power place more importance on the practical value of products, while low-power consumers tend to rely on external cues (e.g., product endorsers) to make decisions and are more attracted to the hedonic value of products, leading to more impulsive consumption behavior (Wang et al., 2023). This finding also supports the idea that individuals with high sense of power focus more on their actual needs and prefer higher-quality, cost-effective tangible products (Rucker and Galinsky, 2009). The study found that people in low-power states preferred larger sizes and brand logos for luxury goods and were more likely to choose status-related luxury brands over mass-market brands (Koo and Im, 2019). This can be explained by compensatory consumption theory, which posits that low-power consumers are more willing to pay a premium for compensatory consumption behaviors (Rucker and Galinsky, 2008). In the context of sports consumption, tangible sports consumption (e.g., sports equipment) and experiential sports consumption (e.g., sports tourism, spectating) differ fundamentally in their value attributes. The former emphasizes ownership and utility, while the latter highlights experiences and emotional engagement (Yu et al., 2025). Based on the Agentic–Communal Model, consumers with high sense of power are likely to prefer tangible sports consumption to meet their functional and self-enhancement needs, while consumers with low sense of power are more inclined toward experiential sports consumption to compensate for psychological deficiencies through social interaction and emotional value (Park et al., 2023). The social nature and conspicuousness of experiential sports consumption further enhance its appeal to individuals with low sense of power. Engaging in leisure sports activities (e.g., outdoor sports, spectating) not only provides opportunities for social connections (Guo et al., 2024) but also alleviates the emotional strain caused by power deficits through the display of experiences. In contrast, purchasing expensive sports products may also signify status but is often associated with materialism, potentially exacerbating the psychological burden of low-power individuals.

*H1: Sense of power significantly negatively affects experiential sports consumption: Consumers with low sense of power are more likely to prefer experiential sports consumption over tangible sports consumption compared to those with high sense of power*.

### 2.3 The mediating role of psychological distance

Psychological distance is an individual's subjective experience of the perceived closeness or distance of something from themselves, typically encompassing three dimensions: temporal, spatial, and social distance (Liberman et al., 2007b). In the context of experiential sports consumption, since the spatial and temporal conditions align with those of companions, the primary focus is on the social distance dimension, which reflects the subjective perception of the closeness or distance in interpersonal relationships (Trope and Liberman, 2010).

Experiential sports consumption is often a group activity, involving a significant amount of interpersonal and social elements. For example, in activities such as hiking, rock climbing, and rafting, close collaboration among participants is required. Compared to going solo, consumers are more willing to engage in group activities, as the happiness derived from shared experiences with others is significantly greater than that from solitary experiences (Caprariello and Reis, 2013). Therefore, a consumer's feelings toward their consumption behavior are closely tied to their evaluation of companions. High-quality companions (those with a closer psychological distance) will lead to a better experience (Li et al., 2021). The Construal Level Theory further explains that when psychological distance changes, an individual's cognition and behavior are also affected accordingly (Weiner, 2000). A reduction in psychological distance causes individuals to feel closer and more connected to others, making communication easier and reducing concerns about interpersonal risks (Hakanson and Ambos, 2010). Thus, in experiential sports consumption, the closer the psychological distance with companions, the higher the consumer's willingness to participate and the more positive the expected experience.

Existing research indicates that the proximity of psychological distance stems from interpersonal differences, with greater interpersonal similarity resulting in closer psychological distance (Bar-Anan et al., 2006). Individuals with a high sense of power tend to have more positive self-assessments, perceiving themselves as superior to others and making lower evaluations of others (Tost et al., 2012). This characteristic leads high-power individuals to rely less on others, demonstrating greater independence, and potentially creating a more distant psychological distance from others (Smith and Trope, 2006). In contrast, individuals with a low sense of power tend to view situations from others' perspectives, have higher empathy, and may therefore have a closer psychological distance to others (Van Kleef et al., 2006). Magee and Smith (2013) further explored the psychological effects of power using Construal Level Theory, suggesting that low-power individuals depend on others to obtain valuable resources, whereas high-power individuals can rely less on others to acquire resources, resulting in a greater social distance for high-power individuals and a closer social distance for low-power individuals (Magee and Smith, 2013).

In summary, the mediating effect of low power leads to a closer psychological distance between individuals and their companions, facilitating closer interactions. This, in turn, enhances the anticipated experience of experiential sports consumption and increases the willingness to engage in such consumption. Based on this, the following research hypothesis is proposed:

*H2: Psychological distance mediates the relationship between sense of power and experiential sports consumption: Compared to high-power individuals, those with low sense of power experience a closer psychological distance with their companions, which increases their willingness to participate in experiential sports consumption*.

### 2.4 The mediating role of social orientation

Social orientation refers to an individual's tendency to actively establish connections and interact with others in social activities (Dholakia et al., 2004). Social orientation strengthens interpersonal social bonds, promotes the exchange of resources, information, and emotions, and satisfies the individual's needs for belonging, intimacy, and trust (Fan et al., 2020), further positively influencing their attitudes and behaviors (Manchanda et al., 2015). Individuals with stronger social orientation are more likely to obtain information, resources, and support through interactions with others (Zhang et al., 2017). Compared to individuals with a high sense of power, those with a low sense of power, due to weaker control over resources, are more reliant on others to compensate for deficiencies (Miyamoto and Ji, 2011), and thus exhibit a stronger social orientation. Additionally, according to the Agentic–Communal Model, high-power individuals tend to be self-interested, focusing on the accumulation of material wealth and social status, while low-power individuals are more communal, emphasizing harmonious social relationships (Rucker and Galinsky, 2016). In summary, individuals with low sense of power (vs. high sense of power) are more likely to exhibit higher social orientation.

In sports activities, participants can build strong social networks by sharing workout methods, results, and experiences (Zhou and Kaplanidou, 2018), which enhances their quality of life and subjective wellbeing (Wei et al., 2025). Empirical research shows that participating in sports activities with others leads to more positive experiences than participating alone (McGowan et al., 2025). Downward and Rasciute (2011) pointed out that socially oriented sports activities significantly enhance participants' happiness levels (Downward and Rasciute, 2011). Studies have also found that when individuals' social needs are unmet, they are more likely to actively seek social interaction and emphasize the social aspects of consumption (Srivastava and Kaul, 2014). For example, in sports spectating, fans establish social connections by discussing event viewpoints, and this interaction creates a unique social experience, significantly enhancing happiness and satisfaction after fulfilling social needs (La Guardia et al., 2000). In contrast, tangible sports consumption (e.g., purchasing sports equipment) focuses more on material possession, with weaker interpersonal interaction and social connection attributes. Therefore, individuals with higher social orientation are more likely to choose experiential sports consumption to meet their social interaction needs.

In conclusion, compared to high-power individuals, low-power individuals, due to their stronger social orientation, are more likely to interact with others through experiential sports consumption, thereby gaining social recognition and alleviating the psychological deficiencies caused by power deficits. Based on this, the following hypothesis is proposed:

*H3: Social orientation mediates the relationship between sense of power and experiential sports consumption: Compared to high-power individuals, low-power individuals are more likely to exhibit higher social orientation, which in turn increases their willingness to participate in experiential sports consumption*.

### 2.5 The chain mediating role of psychological distance and social orientation

In the context of psychological distance and social orientation, it is noted that there is a significant phenomenon of social exclusion between different groups; the greater the psychological distance between groups, the lower their social orientation (Enos and Gidron, 2018). Research also shows that the proximity of interpersonal psychological distance is a key indicator of the quality of social relationships (Zeng et al., 2024). Rachlin and Jones (2008) conducted an interpersonal interaction study and divided participants into in-group and out-group categories, confirming that individuals within the in-group have closer psychological distances and are more likely to engage in helping behaviors, whereas individuals in the out-group have greater psychological distances and are less willing to help others (Rachlin and Jones, 2008). In the context of spectator sports consumption, it is suggested that fans who attend home games experience a sense of “home,” which significantly shortens the psychological distance between them and other spectators, leading to a greater social orientation and enhancing their satisfaction with the spectating experience (Lee et al., 2012).

The Agentic–Communal Model suggests that individuals with an agentic orientation tend to break social norms, and when their sense of agency is threatened, they are more likely to distance themselves from others, showing a negative correlation with social orientation. In contrast, individuals with a communal orientation tend to adhere to social norms, and their communal feelings drive them to seek closer social connections, showing a positive correlation with social orientation (Bartz and Lydon, 2004). Differences in individuals' agentic and communal orientations are closely related to their sense of power: individuals with a high sense of power tend to exhibit an agentic orientation, while those with a low sense of power tend to exhibit a communal orientation (Rucker et al., 2012). Additionally, tangible sports consumption has lower social attributes, which makes it harder for sports consumers to meet their social expectations, thus amplifying negative emotions caused by unmet expectations (Choi et al., 2021). As a result, consumers with a higher social orientation are more likely to choose experiential sports consumption, which offers more social attributes.

Based on the above analysis, we hypothesize that sports consumers with a lower sense of power are more dependent on others, often have closer psychological distances with their companions, and therefore have a higher social orientation. This leads them to be more willing to engage in experiential sports consumption, which satisfies interpersonal interaction needs, rather than in tangible sports consumption. Thus, the following hypothesis is proposed:

*H4: Psychological distance and social orientation mediate the relationship between sense of power and experiential sports consumption in a chain-like manner: Compared to high-power individuals, those with a low sense of power have a closer psychological distance with others and a higher social orientation, which in turn strengthens their willingness to participate in experiential sports consumption*.

**Figure 1:**
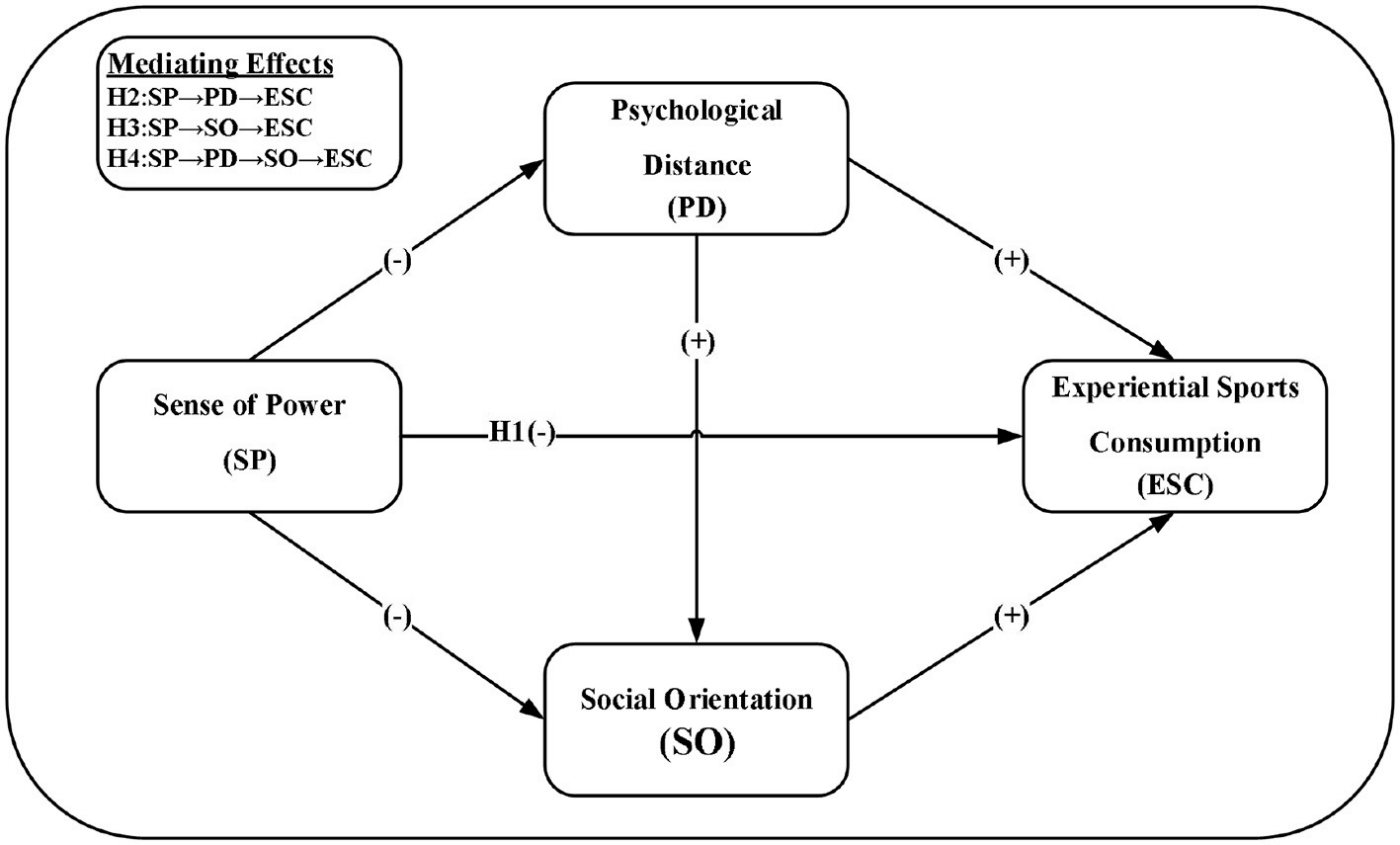
Conceptual model.

In summary, this paper constructs a conceptual model for the impact of sense of power, psychological distance, and social orientation on experiential sports consumption. The specific relationships between variables are illustrated in Figure 1.

## 3 Research design

### 3.1 Research subjects and sample

This study focuses on experiential sports consumers and potential consumers as the research subjects. A combination of convenience sampling and snowball sampling methods was used to collect data. Offline surveys were conducted at large sports consumption events through face-to-face distribution of paper questionnaires. During the questionnaire completion, the researchers provided participants with a detailed explanation of the survey's purpose and the instructions for filling out the questionnaire to ensure the accuracy and validity of the data. After each participant completed the questionnaire, they were given a small gift as a token of appreciation. A total of 468 questionnaires were collected, and after screening and removing incomplete ones, 425 valid questionnaires were obtained, resulting in an effective response rate of 90.8%. Online surveys were conducted via an online survey platform. When participants filled out the electronic questionnaire, the platform provided concise instructions and guidelines to ensure the accuracy of the responses. A total of 715 questionnaires were collected. After screening and eliminating questionnaires that were completed too quickly or contained patterns in responses, 637 valid questionnaires were obtained, yielding an effective response rate of 89.1%. The final sample includes participants from various regions and represents a broad distribution of characteristics such as age, gender, and education level (as detailed in Table 1), making it relatively representative.

**Table 1:** Basic sample information.

**Statistical variable**	**Category**	**Frequency**	**Percentage (%)**
Gender	Male	580	54.6
	Female	482	45.4
Age	Under 25 years	226	21.3
	25–35 years	388	36.5
	36–45 years	291	27.4
	Over 45 years	157	14.8
Education level	High school or below	169	15.9
	Associate degree	322	30.3
	Bachelor's degree	457	43.0
	Master's degree or above	114	10.7
Monthly income	3,000 or below	219	20.6
	3,001–5,000	305	28.7
	5,001–8,000	343	32.3
	Above 8,000	195	18.4
Monthly sports spending	200 or below	477	44.9
	200–500	280	26.4
	500–1,000	195	18.4
	Above 1,000	110	10.4

### 3.2 Variable measurement

The measurement scales used in this study are derived from published and repeatedly validated mature tools to ensure high reliability and validity. To suit the specific consumption context of this study, these scales were revised and finalized through multiple rounds of discussion and revision under the guidance of a group of experts in consumer behavior and sports management. The final formal scales were used to assess four core variables: sense of power, psychological distance, social orientation, and experiential sports consumption. All items were measured using a 7-point Likert scale, where 1 represents “strongly disagree” and 7 represents “strongly agree,” to quantify respondents' subjective attitudes and perceptions. In addition, to ensure the applicability and reliability of the scales used in this study, the following validations were conducted: First, expert reviews were carried out to ensure the content validity of the scales, and a small-scale pilot survey was conducted to verify the clarity of the language and the appropriateness of the item design. Second, reliability analysis (such as Cronbach's α) was used to verify internal consistency, and confirmatory factor analysis (CFA) was conducted to validate the structural validity of the scales.

#### 3.2.1 Sense of power measurement

The sense of power was measured using the Sense of Power Scale (SPS) developed by Anderson et al. (2012), which includes eight items assessing the degree of power in sports consumers, such as “I can make others follow my instructions.” Higher scores indicate a higher sense of power in the sports consumers. Confirmatory factor analysis showed that the standardized factor loadings of all items ranged from 0.762 to 0.853, indicating good construct validity of the scale. The Cronbach's α value was 0.938, demonstrating high internal consistency.

#### 3.2.2 Psychological distance measurement

Psychological distance was measured based on the research by Zeng et al. (2024), using three items to assess the psychological distance between sports consumers and others, such as “I feel very close to my exercise (or viewing) partner.” Higher scores indicate a closer psychological distance. Confirmatory factor analysis showed that the standardized factor loadings of all items ranged from 0.782 to 0.870, indicating good construct validity of the scale. The Cronbach's α value was 0.858, demonstrating high internal consistency.

#### 3.2.3 Social orientation measurement

Social orientation was primarily measured based on the social interaction willingness items from the Motivation Scale for Sport Consumption (MS-SC) developed by Trail and James (2001) and the research findings of Zuo (2020). The scale consists of four items assessing the level of social orientation in sports consumption, such as “In sports consumption (e.g., watching a game), I prefer to be with others rather than alone.” Higher scores indicate a higher social orientation in sports consumers. Confirmatory factor analysis showed that the standardized factor loadings of all items ranged from 0.794 to 0.836, indicating good construct validity of the scale. The Cronbach's α value was 0.885, demonstrating high internal consistency.

#### 3.2.4 Experiential sports consumption measurement

Experiential sports consumption was primarily measured based on the Experiential Buying Tendency Scale (EBTS) developed by Howell et al. (2012) and the Leisure Sports Consumption Willingness Scale developed by Shao et al. (2021). The scale consists of four items assessing the preference for experiential sports consumption, such as “If I have the financial means, I would prefer to purchase sports services (such as sports tourism, attending games, etc.) rather than tangible sports goods (such as sports apparel, etc.).” Except for the reverse-coded item (Item 4), higher scores indicate a higher preference for experiential sports consumption. Confirmatory factor analysis showed that the standardized factor loadings of all items ranged from 0.718 to 0.825, indicating good construct validity of the scale. The Cronbach's α value was 0.860, demonstrating high internal consistency.

## 4 Results and analysis

### 4.1 Common method bias test

This study used anonymous responses and incorporated reverse-coded items and mixed item ordering to reduce common method bias. However, since data were collected using self-reported responses from participants, common method bias could still be present. To address this, the Harman single-factor test was employed to assess all items related to the influence of experiential sports consumption. The results showed that there were four factors with eigenvalues >1. The variance explained by the first factor, without rotation, was 33.63%, which is below the critical threshold of 40%. This indicates that common method bias is acceptable and does not interfere with the subsequent analysis.

### 4.2 Descriptive statistics and correlation analysis

To explore the relationships between sense of power, psychological distance, social orientation, and experiential sports consumption, statistical and correlation analyses were conducted on these variables. The results, shown in Table 2, indicate that sense of power is significantly negatively correlated with psychological distance (γ = −0.513, *P* < 0.01), social orientation (γ = −0.590, *P* < 0.01), and experiential sports consumption (γ = −0.587, *P* < 0.01). Psychological distance is significantly positively correlated with social orientation (γ = 0.458, *P* < 0.01) and experiential sports consumption (γ = 0.520, *P* < 0.01). Social orientation is significantly positively correlated with experiential sports consumption (γ = 0.526, *P* < 0.01). The significant correlations between variables align with the research hypotheses, providing a necessary foundation for subsequent model analysis.

**Table 2:** Descriptive statistics and correlation analysis results of key variables.

**Variables**	**M**	**SD**	**SP**	**PD**	**SO**	**ESC**
SP	3.846	1.489	1			
PD	4.514	1.569	−0.513^**^	1		
SO	4.060	1.359	−0.590^**^	0.458^**^	1	
ESC	4.297	1.519	−0.587^**^	0.520^**^	0.526^**^	1

SP, sense of power; PD, psychological distance; SO, social orientation; ESC, experiential sports consumption. ^**^P < 0.01.

### 4.3 Regression model test

This study uses hierarchical regression analysis to examine the effects of power perception, psychological distance, and social orientation on experiential sports consumption. Hierarchical regression analysis, by progressively introducing different predictor variables, allows for the clarification of each variable's independent contribution after controlling for other factors. This method is particularly suitable for analyzing causal relationships and mediation effects between variables. This study employs this method to explore the direct effect of power perception on sports consumption, as well as the mediating roles of psychological distance and social orientation. To ensure the accuracy and robustness of the regression results, this study includes control variables reflecting demographic characteristics such as gender, age, and education, as well as key consumption-related factors such as monthly income and monthly sports spending. Controlling for these variables helps to eliminate their potential interference with experiential sports consumption, thereby improving the model's validity and the reliability of the results. The final regression model is as follows:


(1) $$\begin{array}{c} Y=\beta_{0}+\sum_{i=j}^{5}\beta_{i}Z_{i}+\beta_{6}X+\beta_{7}M_{1}+\beta_{8}M_{2}+\varepsilon \end{array}$$

In equation (1), *Y* represents the dependent variable experiential sports consumption, *Z*_*i*_ represents the control variables, *X* is the independent variable sense of power, and *M*_1_ and *M*_2_ are the mediator variables psychological distance and social orientation, respectively. β_0_ is the constant term, and β_6_, β_7_, and β_8_ are the regression coefficients, while ε is the error term. By progressively introducing variables, this method helps to reveal how sense of power influences sports consumer behavior through the mediator variables.

The specific results are shown in Table 3. In Model 3, the regression coefficient for sense of power on experiential sports consumption preference was −0.577 (*P* < 0.001), which indicates that the level of the sense of power has a significant negative predictive effect on experiential sports consumption, supporting hypothesis H1. Models 1 and 2 show that the regression coefficients for sense of power on psychological distance and social orientation were −0.498 (*P* < 0.001) and −0.582 (*P* < 0.001), respectively, indicating that the level of sense of power was equally significant in negatively predicting psychological distance and social orientation. Models 4 and 5 show that psychological distance and social orientation have regression coefficients of 0.507 (*P* < 0.001) and 0.511 (*P* < 0.001) on experiential sports consumption preference, respectively, which means that psychological distance and social orientation have a significant positive predictive effect on experiential sports consumption. Furthermore, Models 6, 7, and 8 show that when psychological distance and social orientation variables were introduced separately or simultaneously, the regression coefficients of the sense of power on experiential sports consumption were all weakened, although their predictive effects on experiential sports consumption were still significant. This suggests that psychological distance and social orientation may play a mediating role in the process through which sense of power influences experiential sports consumption preference, providing necessary support for the mediation effect test in the following sections.

**Table 3:** Regression analysis results of the mechanism of sense of power on experiential sports consumption.

**Variables**	**PD**	**SO**	**ESC**					
	**Model 1**	**Model 2**	**Model 3**	**Model 4**	**Model 5**	**Model 6**	**Model 7**	**Model 8**
Gender	0.003	−0.010	−0.018	−0.019	−0.013	−0.019	−0.016	−0.017
Age	−0.017	0.012	−0.038	−0.042	−0.055	−0.033	−0.042	−0.037
Education level	−0.049	−0.027	−0.003	0.052^*^	0.037	0.011	0.004	0.015
Monthly income	0.101^**^	0.046	0.043	0.060^*^	0.077	0.013	0.030	0.007
Monthly sports spending	0.004	0.006	0.030	0.029	0.027	0.028	0.028	0.027
SP	−0.498^***^	−0.582^***^	−0.577^***^			−0.430^***^	−0.418^***^	−0.326^***^
PD				0.507^***^		0.295^**^		0.251^***^
SO					0.511^***^		0.274^***^	0.216^***^
*R* ^2^	0.275	0.351	0.349	0.279	0.287	0.412	0.397	0.441
Adj. *R*^2^	0.271	0.347	0.345	0.275	0.283	0.408	0.393	0.436
*F*	66.703^***^	95.110^***^	94.062^***^	68.101^***^	70.688^***^	105.362^***^	99.229^***^	103.674^***^

^*^P < 0.05, ^**^P < 0.01, ^***^P < 0.001.

### 4.4 Mediation effect test

**Figure 2:**
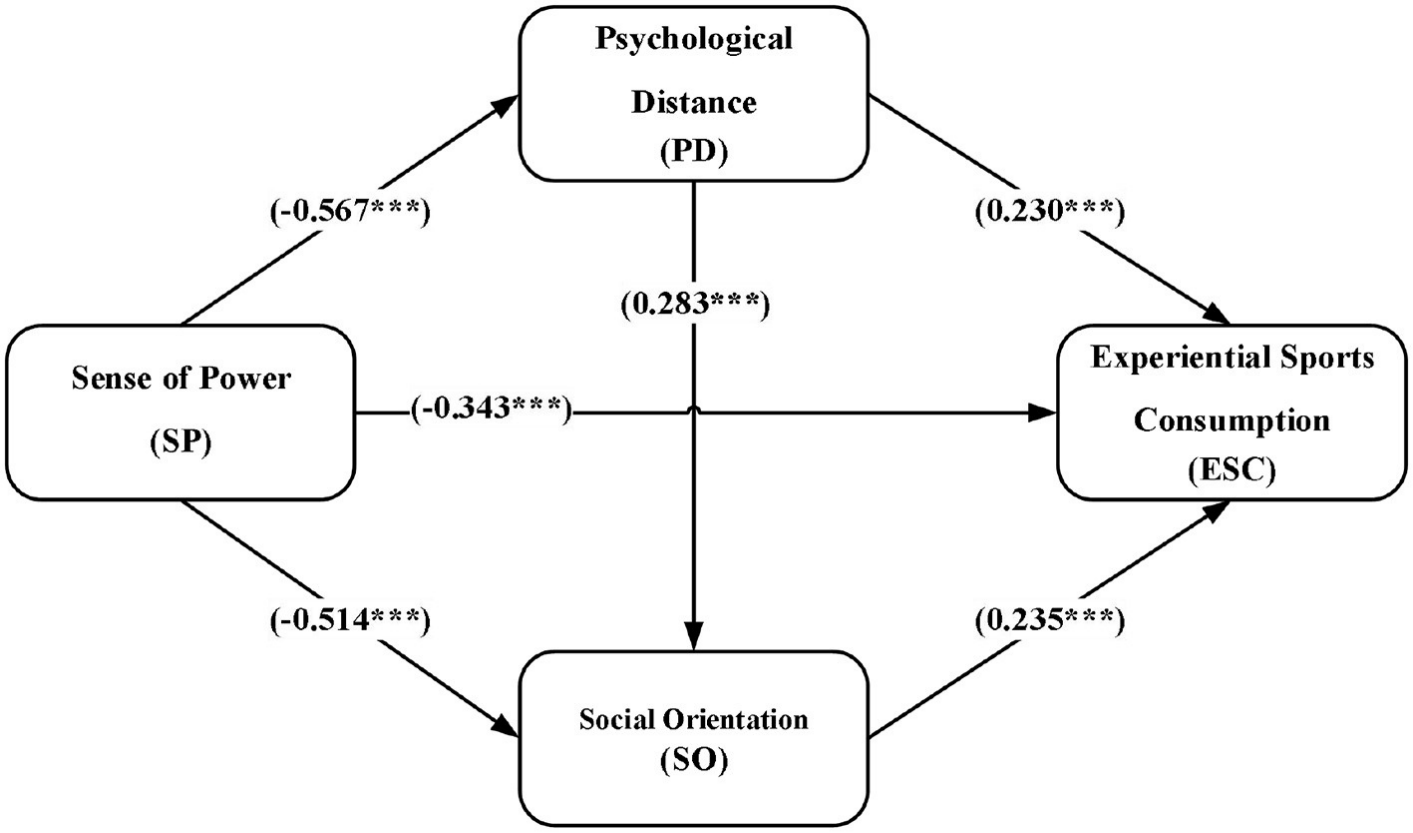
Mediating effect model of psychological distance and social orientation.

A structural equation model was used to establish a relationship model with sense of power as the independent variable, experiential sports consumption as the dependent variable, and psychological distance and social orientation as mediating variables (see Figure 2). The advantage of structural equation modeling (SEM) is to correct measurement errors and test the direct and indirect effects between latent variables simultaneously within a unified analytical framework, so as to accurately reveal the intrinsic structure of theoretical concepts. Using the SEM method, we can clearly analyze the influence mechanism of sense of power on experiential sports consumption through psychological distance and social orientation, and provide a solid statistical basis for the formulation and validation of the mediating hypotheses in this study. The validity of the model was verified by the model fitting goodness of fit (see Table 4). Data analysis showed that: χ^2^/*df* = 1.905, RMR = 0.064, GFI = 0.973, AGFI = 0.965, NFI = 0.979, TLI = 0.988, CFI = 0.990, RMSEA = 0.029. All the values of the indexes meet the threshold requirements in Table 4, indicating that the model fits the data to the ideal degree.

**Table 4:** Validation factor model fit.

**Indicators**	**χ^2^/*df***	**RMR**	**GFI**	**AGFI**	**NFI**	**TLI**	**CFI**	**RMSEA**
Results	1.905	0.064	0.973	0.965	0.979	0.988	0.990	0.029
Standards	< 3	< 0.1	>0.9	>0.9	>0.9	>0.9	>0.9	< 0.05
Situation	Fit	Fit	Fit	Fit	Fit	Fit	Fit	Fit

From Figure 2, it can be seen that the effect of sense of power on experiential sports consumption is a significant negative relationship (γ = −0.343, *P* < 0.001), further confirming hypothesis H1. In addition, sense of power showed a significant negative relationship on both psychological distance (γ = −0.567, *P* < 0.001) and social orientation (γ = −0.514, *P* < 0.001). Psychological distance has significant positive effects on social orientation (γ = 0.230, *P* < 0.001) and experiential sports consumption (γ = 0.283, *P* < 0.001). Social orientation has a significant positive effect on experiential sports consumption (γ = 0.235, *P* < 0.001).

To verify the mediation effects of psychological distance and social orientation in the pathway through which sense of power influences experiential sports consumption, this study used the Bootstrap method to test the significance of the mediation effects using AMOS 24.0 software. A total of 5,000 resamples were conducted with 1,062 samples. If the 95% confidence interval does not contain 0, it indicates the presence of a mediation effect. The Bootstrap analysis for the significance test of the mediation effect is shown in Table 5.

**Table 5:** Mediating effect test of psychological distance and social orientation.

**Path**	**Effect value**	***SE***	***P*-value**	**Bias-corrected 95% CI**		**Mediation percentage (%)**
				**Lower**	**Upper**	
Total effect	−0.655	0.033	^***^	−0.721	−0.592	100
Direct effect	−0.343	0.045	^***^	−0.430	−0.254	52.37
Indirect effect	−0.312	0.035	^***^	−0.380	−0.244	47.63
SP → PD → ESC	−0.160	0.023	^***^	−0.209	−0.117	24.43
SP → SO → ESC	−0.121	0.024	^***^	−0.170	−0.076	18.47
SP → PD → SO → ESC	−0.031	0.007	^***^	−0.047	−0.018	4.73

^***^P < 0.001.

According to Table 5, psychological distance and social orientation both play significant mediating roles between sense of power and experiential sports consumption, with the total indirect effect value being −0.312, accounting for 47.63% of the total effect. Specifically, psychological distance shows a significant mediation effect between sense of power and experiential sports consumption, as the 95% confidence interval does not contain 0, with a standardized effect value of −0.160, accounting for 24.43% of the total effect. This supports hypothesis H2. Social orientation also shows a significant mediation effect between sense of power and experiential sports consumption, as the 95% confidence interval does not contain 0, with a standardized effect value of −0.121, accounting for 18.47% of the total effect. This supports hypothesis H3. Additionally, the chain mediation effect of psychological distance and social orientation between sense of power and experiential sports consumption is significant, as the 95% confidence interval does not contain 0, with a standardized effect value of −0.031, accounting for 4.73% of the total effect. This supports hypothesis H4.

## 5 Discussion

### 5.1 The relationship between sense of power and experiential sports consumption

This study found a significant negative relationship between sports consumers' sense of power and their preference for experiential sports consumption, meaning that individuals with lower sense of power are more inclined to choose experiential sports consumption. This finding can be theoretically interpreted from the dual perspectives of Compensatory Control Theory and the agency-public orientation model. Individuals with a higher sense of power maintain control through resource dominance, and their consumption decisions follow an agency-oriented logic—preferring the certainty of utility in tangible products (such as the visibility of fitness equipment performance) to reinforce their sense of self-efficacy (Rucker et al., 2012). This consumption pattern, dominated by instrumental rationality, is essentially a response to the need to sustain a sense of control (Cutright and Samper, 2014). In contrast, individuals with a lower sense of power, due to weaker resource control, seek social connection compensation through public-oriented behaviors (e.g., group viewing or participation in sports communities; Liu and Mattila, 2017). The associated value of experiential sports consumption (such as the sense of team belonging in event tourism) can alleviate their status anxiety, symbolically compensating for the lack of power.

The present study likewise has some similarities with previous studies (Wang et al., 2023). In earlier studies on savings behavior, it was pointed out that individuals with high sense of power (compared to those with low sense of power) place more value on the instrumental value of money, seeking to accumulate wealth to maintain their existing perception of power and status (Garbinsky et al., 2014). This psychological trait manifests in consumption behavior as individuals with high sense of power focus more on their actual needs and prefer functional, high-quality physical products that emphasize practical value over hedonic value (Rucker and Galinsky, 2009; Wang et al., 2023). Cross-cultural research further indicates that this “material-first” tendency is more pronounced in individualistic cultures, as they place greater emphasis on the material symbolization of personal achievement (Kim and Markus, 1999).

From the perspective of consumption experience, tangible sports consumption (such as purchasing sports equipment or apparel) typically provides short-term happiness, and the satisfaction it brings quickly diminishes over time. To maintain their initial level of happiness, consumers need to continuously upgrade their material consumption. This limits the long-term effect of tangible sports consumption on enhancing happiness. In contrast, experiential sports consumption (such as attending live sports events or paying for fitness sessions) provides intangible emotional experiences, including the pursuit of interests, emotional release, and psychological fulfillment. These experiences not only bring more lasting happiness but also help alleviate the negative emotions caused by power deficits in individuals with lower sense of power (Howell and Hill, 2009). Especially in collectivist cultures, the group affiliation aspect of experiential consumption further enhances its psychological compensatory effect for individuals with a low sense of power (Ianole-Calin et al., 2020).

Furthermore, social comparison theory provides further explanation for this preference difference. Tangible sports products, due to their physical nature, are more likely to be objects of social comparison, potentially triggering negative emotions in low-power consumers, such as inferiority or dissatisfaction. On the other hand, high-power individuals, having a more stable self-concept, are less affected by social comparison. The intangible nature of experiential sports consumption reduces its comparability, allowing low-power consumers to find psychological comfort and avoid the negative effects of social comparison. As a result, individuals with lower sense of power are more likely to choose experiential sports consumption to fulfill their emotional needs and compensate for their lack of power.

### 5.2 The mediating role of psychological distance

Previous research has emphasized the internal perception individuals have regarding the presence or absence of power, or its high or low levels, reflecting the relative dependencies within social relationships (du Plessis et al., 2023). Specifically, individuals with high sense of power typically exhibit confidence, independence, and a sense of control, which leads them to maintain a greater psychological distance from others in social situations (Liu et al., 2024; Rucker et al., 2012). In contrast, individuals with low sense of power, being more dependent on others to achieve their goals, are more attuned to others' needs and tend to reduce interpersonal distance to foster cooperation and resource acquisition (van Kleef et al., 2004).

In the context of sports consumption, this study further found that sense of power in sports consumers has a significant negative relationship with psychological distance, meaning that the higher the sense of power, the greater the psychological distance from others. This result is consistent with prior research (Liu et al., 2024). On the other hand, this study also found that sports consumers with closer psychological distance to others are more likely to prefer experiential sports consumption. Past research has widely confirmed that psychological distance is a key psychological variable determining consumer behavior. Uhm et al. (2022) confirmed in the context of sports e-commerce that augmented reality (AR) experiences can reduce the psychological distance between consumers and products, thereby increasing purchase intentions (Uhm et al., 2022). In experiential sports consumption, the psychological distance between consumers and their companions is particularly important. Zeng et al. (2024) pointed out that satisfaction with experiential consumption is closely related to interpersonal relationships, and companions with closer psychological distance can facilitate more intimate communication, thereby enhancing the quality of the experience (Zeng et al., 2024).

Experiential sports consumption offers consumers diverse opportunities to interact with friends, family, and even strangers. Therefore, the psychological distance between individuals clearly plays a key role in determining their enjoyment of the experience. For example, watching a game with a group of fans supporting the same team naturally shortens the psychological distance between consumers and the group. This sense of closeness enhances consumers' satisfaction with the game and their willingness to continue attending games. In summary, the mediating role of psychological distance between sense of power and experiential sports consumption has been well supported.

### 5.3 The mediating role of social orientation

This study confirms that social orientation is an important psychological factor in predicting experiential sports consumption. This finding is consistent with existing literature, which emphasizes that the socialized nature of experiential consumption is a key factor in its ability to enhance happiness compared to tangible consumption. Specifically, in the field of sports viewing, social interaction motivation has been widely confirmed as a key motivator influencing sports viewing behavior (Lee et al., 2012). For example, Xun et al. (2020) pointed out in their research that spectator sports consumption, as an experiential product, helps sports consumers establish emotional connections with family and friends (Xun et al., 2020). In studies on sports tourism, it has been noted that consumers' social needs with event organizers and other tourists are prerequisites for co-creating value in activities, which can further influence their willingness to participate in sports tourism (Jiang et al., 2021). This study reaches a similar conclusion, namely that social orientation has a significant positive impact on experiential sports consumption.

Furthermore, the study further found that sense of power significantly negatively predicts social orientation in sports consumers. This conclusion is consistent with power control theory and social distance theory: individuals with low sense of power are more sensitive to threats in interpersonal interactions and are thus more likely to alleviate anxiety through social connections. Cai et al. (2023) showed in their study that individuals with high sense of power experience significantly lower loneliness than those with low sense of power, as they are more likely to receive social support; conversely, individuals with low sense of power have unmet belonging needs and exhibit stronger social desires (Cai et al., 2023). In the context of sports consumption, consumers with lower sense of power are more likely to reduce feelings of loneliness through social participation in experiential sports activities, thereby enhancing their social orientation. Compared to tangible sports consumption, experiential sports consumption, with its stronger social interaction component, is better able to meet the psychological needs of individuals with lower sense of power. Zuo (2020) manipulated participants' power status (high vs. low) to measure the impact mechanism between their social orientation and preference for experiential or tangible consumption. The results showed a partial mediation effect of social orientation, indicating that individuals with low sense of power have stronger social orientation and are more inclined toward experiential consumption with social interaction attributes (Zuo, 2020). Thus, the mediating role of social orientation between sense of power and experiential sports consumption has been well supported.

### 5.4 The chain mediating role of psychological distance and social orientation

This study reveals through mediation analysis that psychological distance and social orientation play a chain mediating role between sense of power and experiential sports consumption. Specifically, sports consumers with higher sense of power tend to perceive a greater psychological distance from others. This greater social distance then suppresses their social orientation, ultimately reducing their willingness to engage in experiential sports consumption. This finding provides a new perspective on how sense of power influences sports consumption behavior through psychological and social mechanisms.

Research on power and social cognition has found that individuals' experiences of power increase their level of self-construal and psychological distance from others (Magee and Smith, 2013). In helping behavior research, individuals with high sense of power are less concerned with others' feelings when making resource allocations and value judgments, showing independence and tending to distance themselves from others, which reduces cooperation and prosocial behaviors (Lammers and Stapel, 2011). In contrast, individuals with low sense of power perceive the psychological distance to those they assist as closer, which makes them more likely to exhibit empathy, social connections, and prosocial behaviors (Choi et al., 2012).

Furthermore, as socialized individuals, consumers' behaviors are greatly influenced by the closeness of their group relationships (Liberman et al., 2007a). Research has shown that good interpersonal relationships can facilitate participation in physical activity. For example, in social capital research, a strong social network helps individuals gain support and encouragement, which in turn increases their motivation and frequency of participation in physical activity (Gao et al., 2025). Social orientation is also a strong motivator for individuals to engage in sports activities. Tsai et al. (2021), based on self-determination theory, pointed out that sports applications with social interaction functions can effectively enhance users' motivation and participation in exercise (Tsai et al., 2021).

Related research has highlighted the differences between experiential and tangible sports consumption in terms of social interactions. In experiential sports consumption, many activities are completed in groups and have strong social elements. For example, extreme sports, attending live events, and sports tourism all have significant social attributes, which are more likely to be preferred by individuals with higher social orientation. In summary, sports consumers with a lack of sense of power tend to reduce the psychological distance with others. Closer relationships imply stronger social orientation, which leads them to choose experiential sports consumption behaviors that better satisfy their social needs, compared to tangible sports consumption.

## 6 Conclusion and future outlook

### 6.1 Research conclusions

This study investigates the relationships between sense of power, psychological distance, social orientation, and experiential sports consumption preferences among sports consumers using Structural Equation Modeling (SEM). The results show that sense of power has a significant negative effect on experiential sports consumption preferences (γ = −0.343, *P* < 0.001), meaning that individuals with lower sense of power are more likely to choose sports consumption that is driven by experience and emotion. Sense of power also has a significant negative effect on both psychological distance (γ = −0.567, *P* < 0.001) and social orientation (γ = −0.514, *P* < 0.001), indicating that individuals with lower sense of power tend to engage more in social interactions and maintain closer psychological proximity with others. Psychological distance (γ = 0.283, *P* < 0.001) and social orientation (γ = 0.235, *P* < 0.001) have significant positive effects on experiential sports consumption, suggesting that individuals place more value on social interaction and emotional connection when selecting sports consumption. Furthermore, sense of power indirectly affects experiential sports consumption preferences through psychological distance and social orientation, with psychological distance and social orientation acting as chain mediators between sense of power and experiential sports consumption.

### 6.2 Theoretical contributions

Based on the overview of the research findings, this study makes the following two theoretical contributions:

First, it extends the research on the antecedents of experiential sports consumption. Traditional research on experiential sports consumption often focuses on the attribute differences between experiential and tangible consumption and their differentiated effects on happiness. However, it has rarely explored the deeper factors driving differences in consumption preferences, particularly overlooking psychological antecedents. This study breaks through this limitation by introducing core variables such as sense of power, psychological distance, and social orientation, and systematically analyzing their impact mechanisms on experiential sports consumption. This perspective not only expands the theoretical boundaries of sports consumption behavior but also provides a new research path for revealing the psychological driving factors in consumers' choices between experiential and tangible consumption, enriching the variable system in consumer behavior research.

Second, it reveals the mediating mechanisms of psychological distance and social orientation. This study further proposes and verifies the mediating role of psychological distance and social orientation in the relationship between sense of power and experiential sports consumption, injecting new momentum into the study of psychological mechanisms in sports consumption. The empirical results show that, compared to individuals with high sense of power, low-power consumers are more likely to choose experiential sports consumption due to the closer psychological distance and stronger social orientation they perceive with others. This finding deepens our understanding of how sense of power shapes consumption decisions through social and psychological pathways and resonates with the agency-public orientation model and social distance theory. By constructing a comprehensive framework of how sense of power affects experiential sports consumption, this study not only advances the integration of related theories but also lays a solid foundation for future interdisciplinary research, making a significant theoretical contribution.

### 6.3 Managerial implications

The results of this study provide substantive guidance for sports business managers. First, based on consumer psychological characteristics, it is recommended that sports companies implement refined market segmentation and differentiated product strategies. For high-power consumers, companies should focus on the practical value of products, highlighting the functionality, performance advantages, and durability of sports products to meet their preference for high-quality tangible goods. For low-power consumers, companies should emphasize the emotional and social value of experiences, designing participatory sports activities such as group events or sports programs enhanced by social interaction to improve emotional satisfaction and meet social needs, thereby increasing market attractiveness and marketing efficiency.

Furthermore, it is recommended that sports companies focus service design on reducing the psychological distance between consumers and enhancing social interaction. For example, offering couple or family sports programs or creating environments that support social interaction at sports events, such as setting up exclusive areas for home and away teams or providing team-themed apparel, can promote connections and group identity among participants. These measures will not only enhance the overall experience quality for consumers but also help strengthen brand loyalty and the long-term potential for value creation through positive feedback mechanisms from social interactions.

### 6.4 Limitations and future outlook

This study has several limitations, which also provide directions for future research. First, the study only analyzes the main effects of sense of power differences and their mediating role through psychological distance and social orientation. However, the complexity of experiential sports consumption suggests that other influencing factors may exist, particularly the heterogeneity of the social attributes across different consumption activities, which may interfere with the conclusions. For example, team sports activities (such as sports clubs or group projects) are highly social, whereas independent activities (such as solo workouts or online sports viewing) are less social. This difference may moderate the relationship between sense of power and consumer behavior. Future research should explore the diversity effect of consumption activities to enhance the generalizability of the theory. Second, the sample in this study comes from China, and a single cultural background may influence the results, particularly the differences between collectivist and individualist cultures. Future research should consider comparative analysis across different cultural contexts to validate the impact of cultural differences on consumer behavior. Third, this study focuses on experiential sports consumption and does not fully consider the interaction between tangible and experiential consumption. For example, outdoor adventure activities involve both experiential consumption (such as guided tours) and tangible consumption (such as equipment purchase), with both factors jointly influencing consumer decisions. Future research should construct an integrative model to explore the synergistic effects of these two forms of consumption and their combined influence. Fourth, the cross-sectional design and convenience sampling used in this study may limit the robustness of the findings. Cross-sectional data are difficult to reveal dynamic causal relationships among variables, and the long-term interaction effect between sense of power and experiential sports consumption needs to be verified through longitudinal tracking studies; convenience sampling and snowball sampling may lead to group coverage bias, which can be combined with stratified random sampling and broader demographic characteristics to enhance generalizability in the future.

## Data Availability

The original contributions presented in the study are included in the article/Supplementary material, further inquiries can be directed to the corresponding authors.

## References

[B1] Anderson C. John O. P. Keltner D. (2012). The personal sense of power. J. Pers. 80, 313–344. 10.1111/j.1467-6494.2011.00734.x 21446947

[B2] Bar-Anan Y. Liberman N. Trope Y. (2006). The association between psychological distance and construal level: evidence from an implicit association test. J. Exp. Psychol. General 135, 609–622. 10.1037/0096-3445.135.4.609 17087576

[B3] Bartz J. A. Lydon J. E. (2004). Close relationships and the working self-concept: implicit and explicit effects of priming attachment on agency and communion. Pers. Soc. Psychol. Bull. 30, 1389–1401. 10.1177/0146167204264245 15448304

[B4] Cai W. Guinote A. Wu S. (2023). Revisiting the powerful-not-lonely effect across cultures: the mediating role of self-construal and social support. Curr. Psychol. 42, 8824–8832. 10.1007/s12144-021-02157-w

[B5] Caprariello P. A. Reis H. T. (2013). To do, to have, or to share? Valuing experiences over material possessions depends on the involvement of others. J. Pers. Soc. Psychol. 104, 199–215. 10.1037/a0030953 23276272

[B6] Chanavat N. Bodet G. (2014). Experiential marketing in sport spectatorship services: a customer perspective. Euro. Sport Manage. Q. 14, 323–344. 10.1080/16184742.2014.926379

[B7] Choi S. Mattila A. S. Bolton L. E. (2021). To err is human(-oid): how do consumers react to robot service failure and recovery? J. Service Res. 24, 354–371. 10.1177/1094670520978798

[B8] Choi S. Y. Park H. S. Oh J. Y. (2012). Temporal distance and blood donation intention. J. Health Psychol. 17, 590–599. 10.1177/1359105311421048 21914769

[B9] Cutright K. M. Samper A. (2014). Doing it the hard way: how low control drives preferences for high-effort products and services. J. Consumer Res. 41, 730–745. 10.1086/677314

[B10] Dholakia U. M. Bagozzi R. P. Pearo L. K. (2004). A social influence model of consumer participation in network- and small-group-based virtual communities. Int. J. Res. Market. 21, 241–263. 10.1016/j.ijresmar.2003.12.004

[B11] Downward P. Rasciute S. (2011). Does sport make you happy? An analysis of the well-being derived from sports participation. Int. Rev. Appl. Econ. 25, 331–348. 10.1080/02692171.2010.511168

[B12] du Plessis C. Nguyen M. H. B. Foulk T. A. Schaerer M. (2023). Relative power and interpersonal trust. J. Pers. Soc. Psychol. 124, 567–592. 10.1037/pspi0000401 35816569

[B13] Dunn E. W. Gilbert D. T. Wilson T. D. (2011). If money doesn't make you happy, then you probably aren't spending it right. J. Consumer Psychol. 21, 115–125. 10.1016/j.jcps.2011.02.002

[B14] Enos R. D. Gidron N. (2018). Exclusion and cooperation in diverse societies: experimental evidence from Israel. Am. Polit. Sci. Rev. 112, 742–757. 10.1017/S0003055418000266

[B15] Fan Y. Jiang J. Hu Z. (2020). Abandoning distinctiveness: the influence of nostalgia on consumer choice. Psychol. Market. 37, 1342–1351. 10.1002/mar.21370

[B16] Gao Z. Chee C. S. Dev R. D. O. Liu Y. Gao J. Li R.. (2025). Social capital and physical activity: a literature review up to March 2024. Front. Public Health 13:1467571. 10.3389/fpubh.2025.1467571 40013056 PMC11860974

[B17] Garbinsky E. N. Klesse A.-K. Aaker J. (2014). Money in the bank: feeling powerful increases saving. J. Consumer Res. 41, 610–623. 10.1086/676965

[B18] Gilovich T. Kumar A. Jampol L. (2015). The beach, the bikini, and the best buy: replies to Dunn and Weidman, and to Schmitt, Brakus, and Zarantonello. J. Consumer Psychol. 25, 179–184. 10.1016/j.jcps.2014.09.002

[B19] Guinote A. (2017). How power affects people: activating, wanting, and goal seeking. Annu. Rev. Psychol. 68, 353–381. 10.1146/annurev-psych-010416-044153 27687123

[B20] Guo J. Yang H. Zhang X. (2024). How watching sports events empowers people's sense of wellbeing? The role of chain mediation in social interaction and emotional experience. Front. Psychol. 15:1471658. 10.3389/fpsyg.2024.1471658 39712543 PMC11659752

[B21] Guo K. Wang Z. Li C. Guo C. (2023). Meaningful sports consumption behavior influences the production of individual eudaimonic well-being: the chain-mediated role of perceived social support and perceived self-esteem. Sustainability 15:14043. 10.3390/su151914043

[B22] Hadi R. Melumad S. Park E. S. (2024). The metaverse: a new digital frontier for consumer behavior. J. Consumer Psychol. 34, 142–166. 10.1002/jcpy.1356

[B23] Hakanson L. Ambos B. (2010). The antecedents of psychic distance. J. Int. Manage. 16, 195–210. 10.1016/j.intman.2010.06.001

[B24] Hamari J. Sjoblom M. (2017). What is eSports and why do people watch it? Internet Res. 27, 211–232. 10.1108/IntR-04-2016-0085

[B25] Howell R. T. Hill G. (2009). The mediators of experiential purchases: determining the impact of psychological needs satisfaction and social comparison. J. Positive Psychol. 4, 511–522. 10.1080/17439760903270993

[B26] Howell R. T. Pchelin P. Iyer R. (2012). The preference for experiences over possessions: measurement and construct validation of the Experiential Buying Tendency Scale. J. Positive Psychol. 7, 57–71. 10.1080/17439760.2011.626791

[B27] Ianole-Calin R. Francioni B. Masili G. Druica E. Goschin Z. (2020). A cross-cultural analysis of how individualism and collectivism impact collaborative consumption. Resourc. Conserv. Recycl. 157:104762. 10.1016/j.resconrec.2020.104762

[B28] Jeon Y. Kim D. Han S. Huang Y. Kim J. (2021). How does service environment enhance consumer loyalty in the sport fitness industry? The role of servicescape, cosumption motivation, emotional and flow experiences. Sustainability 13:6414. 10.3390/su13116414

[B29] Jiang X. Kim A. Kim K. Yang Q. Garcia-Fernandez J. Zhang J. J. (2021). Motivational antecedents, value co-creation process, and behavioral consequences in participatory sport tourism. Sustainability 13:9916. 10.3390/su13179916

[B30] Kim H. Markus H. R. (1999). Deviance or uniqueness, harmony or conformity? A cultural analysis. J. Pers. Soc. Psychol. 77:785. 10.1037/0022-3514.77.4.785

[B31] Kim J. James J. D. (2019). Sport and happiness: understanding the relations among sport consumption activities, long- and short-term subjective well-being, and psychological need fulfillment. J. Sport. Manage. 33, 119–132. 10.1123/jsm.2018-0071

[B32] Koo J. Im H. (2019). Going up or down? Effects of power deprivation on luxury consumption. J. Retail. Consumer Serv. 51, 443–449. 10.1016/j.jretconser.2017.10.017

[B33] La Guardia J. G. Ryan R. M. Couchman C. E. Deci E. L. (2000). Within-person variation in security of attachment: a self-determination theory perspective on attachment, need fulfillment, and well-being. J. Pers. Soc. Psychol. 79, 367–384. 10.1037/0022-3514.79.3.367 10981840

[B34] Lammers J. Stapel D. A. (2011). Power increases dehumanization. Group Process. Intergr. Relat. 14, 113–126. 10.1177/1368430210370042

[B35] Lee J. C. Hall D. L. Wood W. (2018). Experiential or material purchases? Social class determines purchase happiness. Psychol. Sci. 29, 1031–1039. 10.1177/0956797617736386 29792773

[B36] Lee S. Lee H. J. Seo W. J. Green C. (2012). A new approach to stadium experience: the dynamics of the sensoryscape, social interaction, and sense of home. J. Sport. Manage. 26, 490–505. 10.1123/jsm.26.6.490

[B37] Li C. Qu W. (2025). The impact of social exclusion on experiential sports consumption: the chain mediating roles of loneliness and the need for social connection. Front. Psychol. 16:1532643. 10.3389/fpsyg.2025.1532643 39944043 PMC11814200

[B38] Li Q. Fan Y. Song W. (2021). The influence of social context on the engagement interest in experiential purchase. Nankai Business Rev. 24, 4–17.

[B39] Li Y. Kou X. Li Z. Ye S. (2025). Dynamic mechanism and evolutionary game analysis of sports industry service transformation. Sci. Rep. 15:3381. 10.1038/s41598-025-88026-z 39870861 PMC11772834

[B40] Liberman N. Trope Y. McCrea S. M. Sherman S. J. (2007b). The effect of level of construal on the temporal distance of activity enactment. J. Exp. Soc. Psychol. 43, 143–149. 10.1016/j.jesp.2005.12.009

[B41] Liberman N. Trope Y. Wakslak C. (2007a). Construal level theory and consumer behavior. J. Consumer Psychol. 17, 113–117. 10.1016/S1057-7408(07)70017-7 PMC3150814 21822366

[B42] Liu S. Q. Mattila A. S. (2017). Airbnb: online targeted advertising, sense of power, and consumer decisions. Int. J. Hospital. Manage. 60, 33–41. 10.1016/j.ijhm.2016.09.012

[B43] Liu Z. Luan M. Li H. Stoker J. I. Lammers J. (2024). Psychological power increases the desire for social distance but reduces the sense of social distance. J. Exp. Soc. Psychol. 110:104528. 10.1016/j.jesp.2023.104528

[B44] Magee J. C. Galinsky A. D. (2008). 8 social hierarchy: the self-reinforcing nature of power and status. Acad. Manag. Ann. 2, 351–398. 10.5465/19416520802211628

[B45] Magee J. C. Smith P. K. (2013). The social distance theory of power. Pers. Soc. Psychol. Rev. 17, 158–186. 10.1177/1088868312472732 23348983

[B46] Manchanda P. Packard G. Pattabhiramaiah A. (2015). Social dollars: the economic impact of customer participation in a firm-sponsored online customer community. Market. Sci. 34, 367–387. 10.1287/mksc.2014.089019642375

[B47] Matte J. Fachinelli A. C. De Toni D. Milan G. S. Olea P. M. (2024). Relationship between leisure involvement, voluntary simplicity, leisure satisfaction, and experiential consumption. Leisure Sci. 46, 512–531. 10.1080/01490400.2021.2001703

[B48] McGowan L. J. Davies A. French D. P. Devereux-Fitzgerald A. Boulton E. Todd C.. (2025). Understanding the experiences of older adult participants and individuals involved in the delivery of a physical activity programme based on participatory approaches: a qualitative analysis. Br. J. Health Psychol. 30:e12747. 10.1111/bjhp.12747 39313443 PMC11586820

[B49] Miyamoto Y. Ji L.-J. (2011). Power fosters context-independent, analytic cognition. Pers. Soc. Psychol. Bull. 37, 1449–1458. 10.1177/0146167211411485 21653580

[B50] Neumann D. L. Moffitt R. L. Thomas P. R. Loveday K. Watling D. P. Lombard C. L.. (2018). A systematic review of the application of interactive virtual reality to sport. Virtual Real. 22, 183–198. 10.1007/s10055-017-0320-5

[B51] Nicolao L. Irwin J. R. Goodman J. K. (2009). Happiness for sale: do experiential purchases make consumers happier than material purchases? J. Consumer Res. 36, 188–198. 10.1086/597049

[B52] Park S. Kim C. Park J. (2023). The interplay between perceived busyness and power distance belief on subjective social status: consequences for consumer preferences for material versus experiential purchases. J. Consumer Behav. 22, 1462–1473. 10.1002/cb.2229

[B53] Rachlin H. Jones B. A. (2008). Altruism among relatives and non-relatives. Behav. Processes 79, 120–123. 10.1016/j.beproc.2008.06.002 18625292 PMC2561243

[B54] Raghunathan R. Corfman K. (2006). Is happiness shared doubled and sadness shared halved? Social influence on enjoyment of hedonic experiences. J. Market. Res. 43, 386–394. 10.1509/jmkr.43.3.38611670861

[B55] Rosenzweig E. Gilovich T. (2012). Buyer's remorse or missed opportunity? Differential regrets for material and experiential purchases. J. Pers. Soc. Psychol. 102, 215–223. 10.1037/a0024999 21843013

[B56] Rucker D. D. Dubois D. Galinsky A. D. (2011). Generous paupers and stingy princes: power drives consumer spending on self versus others. J. Consumer Res. 37, 1015–1029. 10.1086/657162

[B57] Rucker D. D. Galinsky A. D. (2008). Desire to acquire: powerlessness and compensatory consumption. J. Consumer Res. 35, 257–267. 10.1086/588569

[B58] Rucker D. D. Galinsky A. D. (2009). Conspicuous consumption versus utilitarian ideals: how different levels of power shape consumer behavior. J. Exp. Soc. Psychol. 45, 549–555. 10.1016/j.jesp.2009.01.005

[B59] Rucker D. D. Galinsky A. D. (2016). The agentic-communal model of power: implications for consumer behavior. Curr. Opin. Psychol. 10, 1–5. 10.1016/j.copsyc.2015.10.010

[B60] Rucker D. D. Galinsky A. D. Dubois D. (2012). Power and consumer behavior: how power shapes who and what consumers value. J. Consumer Psychol. 22, 352–368. 10.1016/j.jcps.2011.06.001

[B61] Shao X. Qiu L. Zhang Q. Tian Y. (2021). Influence of leisure sports consumption motivation on behavior will: double mediating effect of leisure involvement and experience quality. J. Xi'an Phys. Educ. Univ. 38, 174–225. 10.16063/j.cnki.issn1001-747x.2021.02.008

[B62] Smith P. K. Trope Y. (2006). You focus on the forest when you're in charge of the trees: power priming and abstract information processing. J. Pers. Soc. Psychol. 90, 578–596. 10.1037/0022-3514.90.4.578 16649856

[B63] Song X. Hou J. Li Z. Liu N. (2023). Having or lacking power leads to impulse buying? The influence of power and buying impulsiveness trait on impulse buying. J. Psychol. Sci. 46, 1188–1195. 10.16719/j.cnki.1671-6981.20230520

[B64] Srivastava M. Kaul D. (2014). Social interaction, convenience and customer satisfaction: the mediating effect of customer experience. J. Retail. Consumer Serv. 21, 1028–1037. 10.1016/j.jretconser.2014.04.00736661588

[B65] Tost L. P. Gino F. Larrick R. P. (2012). Power, competitiveness, and advice taking: why the powerful don't listen. Organ. Behav. Hum. Decis. Process. 117, 53–65. 10.1016/j.obhdp.2011.10.001

[B66] Trail G. T. James J. D. (2001). The motivation scale for sport consumption: assessment of the scale's psychometric properties. J. Sport Behav. 24, 108–127.27409075

[B67] Trope Y. Liberman N. (2010). Construal-level theory of psychological distance. Psychol. Rev. 117, 440–463. 10.1037/a0018963 20438233 PMC3152826

[B68] Tsai T.-H. Chang Y.-S. Chang H.-T. Lin Y.-W. (2021). Running on a social exercise platform: applying self-determination theory to increase motivation to participate in a sporting event. Comput. Human Behav. 114:106523. 10.1016/j.chb.2020.106523

[B69] Uhm J.-P. Kim S. Do C. Lee H.-W. (2022). How augmented reality (AR) experience affects purchase intention in sport E-commerce: roles of perceived diagnosticity, psychological distance, and perceived risks. J. Retail. Consumer Serv. 67:103027. 10.1016/j.jretconser.2022.103027

[B70] Van Boven L. Gilovich T. (2003). To do or to have? That is the question. J. Pers. Soc. Psychol. 85, 1193–1202. 10.1037/0022-3514.85.6.1193 14674824

[B71] van Kleef G. A. De Dreu C. K. W. Manstead A. S. R. (2004). The interpersonal effects of anger and happiness in negotiations. J. Pers. Soc. Psychol. 86, 57–76. 10.1037/0022-3514.86.1.57 14717628

[B72] Van Kleef G. A. De Dreu C. K. W. Pietroni D. Manstead A. S. R. (2006). Power and emotion in negotiation: power moderates the interpersonal effects of anger and happiness on concession making. Eur. J. Soc. Psychol. 36, 557–581. 10.1002/ejsp.320

[B73] Wang F. Zhou J. Fan C. (2024). Exploring the factors influencing public intention for spectator sports consumption based on grounded theory. Sci. Rep. 14:8221. 10.1038/s41598-024-59049-9 38589500 PMC11001854

[B74] Wang Y. Yao T. Qiu Q. (2023). From experience to expectation: the reverse effect of power on purchasing impulsiveness. Front. Psychol. 14:1094536. 10.3389/fpsyg.2023.1094536 36968728 PMC10032043

[B75] Wei P. Q. Qin T. Zhu C. Y. (2025). Effects of physical activity participation on subjective well-being of Chinese residents: mediating effects of physical health status and perceived social development. Front. Psychol. 16:1415158. 10.3389/fpsyg.2025.1415158 39917735 PMC11798977

[B76] Weiner B. (2000). Intrapersonal and interpersonal theories of motivation from an attributional perspective. Educ. Psychol. Rev. 12, 1–14. 10.1023/A:1009017532121

[B77] Wu C. (2024). The impact of sports industry output on economic growth: evidence from China. J. Knowl. Econ. 1–21. 10.1007/s13132-024-02218-y

[B78] Xun Y. Song L. Huang Q. Cao M. Ge X. (2020). Research on the influence mechanism of show-oriented sports consumer loyalty under the product involvement. J. Xi'an Phys. Educ. Univ. 37, 696–704. 10.16063/j.cnki.issn1001-747x.2020.06.008

[B79] Yu D. Fan H. Zhang N. (2025). Factors influencing fans' spectating experience and configuration effects in CBA league. PLoS ONE 20:e0316706. 10.1371/journal.pone.0316706 39752398 PMC11698371

[B80] Zeng F. Liu M. Chi Y. Jin Q. (2024). Only wishing for “one person”: a study on the impact of companion numbers on the willingness to purchase public hedonic experiential consumption. Nankai Business Rev. 27, 185–196.

[B81] Zhang C. Phang C. W. Wu Q. S. Luo X. M. (2017). Nonlinear effects of social connections and interactions on individual goal attainment and spending: evidences from online gaming markets. J. Mark. 81, 132–155. 10.1509/jm.16.003811670861

[B82] Zhang J. Beatty S. E. Walsh G. (2008). Review and future directions of cross-cultural consumer services research. J. Bus. Res. 61, 211–224. 10.1016/j.jbusres.2007.06.003

[B83] Zhao Y. Jin X. (2021). The effect of loneliness on experiential consumption preference. China Business Market 35, 100–109. 10.14089/j.cnki.cn11-3664/f.2021.02.00933439127

[B84] Zheng J. Shen J. (2025). Social comparisons for well-being: the role of power distance. Soc. Indicat. Res. 1–24. 10.1007/s11205-025-03531-y

[B85] Zhou R. Kaplanidou K. (2018). Building social capital from sport event participation: an exploration of the social impacts of participatory sport events on the community. Sport Manage. Rev. 21, 491–503. 10.1016/j.smr.2017.11.001

[B86] Zuo X. (2020). Research on the effect of power state on consumers' preference for experiential or material consumption (PhD). Available online at: https://link.cnki.net/doi/10.27162/d.cnki.gjlin.2020.007332 (accessed December 17, 2024).

